# A framework for assessing forest habitat connectivity loss and optimising reforestation efforts on the example of a hydropower project

**DOI:** 10.1007/s00267-025-02351-7

**Published:** 2025-12-27

**Authors:** Federica Fonda, Maria Petrillo, Giovanni Bacaro

**Affiliations:** 1 Department of Life Science, University of Trieste, Trieste, Italy; 2 Department of Agricultural, Food and Forest Sciences (SAAF), University of Palermo, Viale delle Scienze, Palermo, Italy

**Keywords:** Environmental Impact Assessment, Hydroelectric, Reservoir, Terrestrial habitat, Offset, Ecological connectivity

## Abstract

The development of renewable energy, such as hydropower, often leads to the loss and fragmentation of terrestrial habitats, with significant effects on biodiversity. However, these impacts are often overlooked or underestimated in Environmental Impact Assessments (EIAs). In this study, we proposed a framework that explicitly integrates forest habitat connectivity into EIAs and environmental planning, addressing both impact assessment and recommendations for offset. The framework included (i) an evaluation of forest habitat connectivity loss by comparing before- and after-construction scenarios and (ii) a spatial analysis to prioritise reforestation activities aimed at restoring connectivity for wildlife species with varying movement abilities. We applied this framework to a proposed hydropower project in Nepal and found a major loss of forest habitat connectivity within the project footprint and up to 15 km away, indicating substantial loss of forest connectivity and a landscape-scale impact. In total, 94.1 km^2^ of deforested areas were identified, and potential reforestation sites were ranked using the Integral Index of Connectivity across three dispersal distances (500 m, 1 km and 2 km). Priority reforestation sites were identified both at landscape-level and within riparian zones, with 15% and 36% of the sites, respectively, emerging as top priorities for reforestation across all the dispersal distances. Our findings highlight the importance of explicitly addressing habitat connectivity in EIAs and environmental planning and show how connectivity-based prioritisation can guide effective reforestation offsets, promoting a better balance between energy development and biodiversity conservation.

## Introduction

The transition to renewable energy production is indispensable for reducing greenhouse gas (GHG) emissions and limiting global warming to 1.5 °C, as targeted in the Paris Agreement (2015). The world’s largest sources of renewable electricity generation—hydro, solar and wind—have low GHG emissions, and according to the IEA (2024), global renewable capacity is expected to grow 2.7 times by 2030. While hydropower remains the largest and most extensively deployed renewable energy source, its importance is expected to grow less than that of solar and wind (IEA 2024). Nevertheless, hydropower is anticipated to expand further, particularly in emerging and developing countries, as new projects are planned and commissioned (Moran et al. 2018; IEA 2024). Hydropower and other renewable energy sources require considerable financial investment and offer substantial energy generation potential. However, they also raise critical environmental concerns and pose complex challenges related to biodiversity conservation (Gibson et al. 2017; Moran et al. 2018). Among renewables, reservoir-based hydropower has the greatest environmental impacts (Gibson et al. 2017), mainly due to the loss and fragmentation of habitats. Hydropower reservoirs typically require extensive land areas for water storage and flow regulation, often leading to substantial landscape transformation (Gibson et al. 2017). Most of the scientific literature has focused on the impacts of hydropower on freshwater ecosystems (Winemiller et al. 2016; Japoshvili et al. 2021), riverine biodiversity (He et al. 2024) and mitigation measures for fish species (Haraldstad et al. 2019; Zarri et al. 2022). In contrast, although land use change has been considered as a major impact of hydropower development (Gracey and Verones 2016), deforestation of natural, biodiversity-rich areas has been documented (Pandit and Grumbine 2012) and impacts on habitats of terrestrial species have been recognized (Palmeirim and Gibson 2021), relatively little attention has been paid to terrestrial habitats and their connectivity, even though impacts can be relevant (Pandit and Grumbine 2012; Gracey and Verones 2016; Dorber et al. 2020). Ecological connectivity, which is defined as “the unimpeded movement of species and the flow of natural processes that sustain life on Earth” (CMS 2020), plays a critical role in long-term conservation strategies. It is essential for maintaining viable populations, supporting gene flow and allowing species to move and to adapt to environmental changes (Fischer and Lindenmayer 2007; Hilty et al. 2020). Ecological connectivity can be distinguished into structural and functional components (Hilty et al. 2019). Structural connectivity refers to physical arrangement of habitats across the landscape, and it often aims to identify possible corridors that may facilitate species movement (Forman and Godron 1986; Hilty et al. 2020). Functional connectivity, on the other hand, reflects how well genes, gametes, propagules or individuals can actually move and disperse across the landscape (Hilty et al. 2020).

While connectivity impacts of hydropower have been studied in rivers (Finer and Jenkins 2012; Anderson et al. 2018), its effects on forest habitat connectivity remain underrepresented in Environmenta Impact Assessments (EIAs), where forest fragmentation is often not quantitatively assessed. Forest fragmentation isolates forest patches and exacerbates edge effect, disrupting habitats and altering biotic and abiotic conditions, and favours the introduction of invasive alien species (Fahrig 2003). These changes alter wildlife movement, reduce habitat quality and affect ecological networks. Dorber et al. (2023) demonstrated the impact of a hydropower project in Norway on the connectivity of the migratory wild reindeer (*Rangifer tarandus tarandus*). Furthermore, they found that the cumulative effect of hydropower and associated infrastructure extends far beyond the reservoir, affecting the entire ecological network of reindeer. Similarly, Lan et al. (2024) found that reservoirs in the Indochina Peninsula altered habitat connectivity and exacerbated habitat fragmentation for terrestrial mammals. These examples, among the others, clearly confirm the negative effect of reservoir-based hydropower projects on terrestrial habitat connectivity. In addition, hydropower projects can exert considerable threat on riverine forest (Resende et al. 2019; He et al. 2024), which are particularly sensitive habitats (Larsen-Gray and Loehle 2022). The creation of reservoir and associated infrastructure often leads to the direct loss of riparian vegetation through inundation, while altering hydrological regimes modify natural flooding and sedimentation that are essential for riverine forest (Nilsson and Berggren 2000).

The lack of consideration of connectivity in EIAs is not a new issue (Fonda et al. 2026; Patterson et al. 2022; Torres et al. 2022). Although many connectivity studies have been published and a wide range of connectivity metrics and modelling techniques are available (Hilty et al. 2020; Keeley et al. 2021; CCSG 2024), their practical applications in EIAs remains limited (Torres et al. 2022). The main reasons appear to be related to difficulties in quantitative assessment of connectivity and inadequate guidance and national/international legislation (Patterson et al. 2022; Torres et al. 2022). EIAs play a pivotal role in sustainable development, including renewable energy projects (Dorber et al. 2023), and must identify and predict all possible environmental impacts of a proposed project. In addition to their legal and procedural roles, EIAs often embedded within broader and international sustainability frameworks that aim to safeguard biodiversity, such as the International Finance Corporation, Performance Standard 6 (IFC 2019). This standard requires that projects demonstrate a pathway towards achieving *no net loss* of biodiversity, and in some cases *net gain* (IFC 2019). This is implemented through the mitigation hierarchy, which is a sequential process that prioritises the avoidance of the impact first, followed by minimisation, restoration and, if necessary, offsetting of residual impacts (IFC 2019; Bennun et al. 2021). Within this framework, the term “offset” carries a specific meaning, referring to measurable and ecologically demonstrable gains that balance residual impacts after all other mitigation steps have been applied. This distinguishes offsets from generic compensation measures, which may not be required to achieve ecological equivalence or additionality. Adopting this terminology is therefore essential when discussing reforestation or restoration actions intended to contribute explicitly to biodiversity outcomes, in line with internationally recognised standards such as those developed under (BBOP 2009).

The mitigation hierarchy is widely recognised as a key approach for biodiversity management in development project, providing a framework to balance infrastructure development with conservation goals. In this context, habitat connectivity plays a critical role, as fragmentation and connectivity loss can affect each stage of mitigation hierarchy. For example, avoiding impacts becomes difficult if corridors are not identified, restoration efforts or offsets may be ineffective if some patches remain isolated and connectivity is not properly incorporated. Thus, while impact assessments on habitat connectivity are often overlooked, their inclusion in EIAs are essential to ensure the effectiveness of mitigation and offsetting efforts in hydropower and large-scale projects (Bergès et al. 2020).

Here, we propose the application of a framework to explicitly include the impact of reservoir-based hydropower on forest habitat connectivity in EIA. This study aims to assess the changes in forest habitat connectivity resulting from the construction of a proposed hydropower project in Nepal, and to support reforestation activities as a offset committed in the EIA designed to restore and enhance connectivity after project implementation. Nepal, a developing country and a globally recognized biodiversity hotspot (Mittermeier et al. 2011), along with other Himalayan countries, is experiencing a rapid expansion of hydropower development (Pandit and Grumbine 2012; Dhyani 2023; NEA 2024). This is largely due to the mountainous environment of the region, which is particularly suitable for this type of project and represents a good example to apply the framework of this study. Indeed, in Nepal, all large infrastructure projects, including hydropower above 50 MV, are legally required to conduct a full EIA under the *Environment Protection Act (2019)* and *Environment Protection Regulation (2020)*. These assessments are not only designed to identify and predict potential impacts, but also to ensure that effective mitigation measures are implemented. When forest land is cleared for development, project developers are obliged to compensate through reforestation activities (Shrestha and Praveen 2024). A distinctive feature of Nepal’s approach is the involvement of community forest institutions, which manage a large portion of the government country’s forest and play central role in planning and implementation of reforestation activities (Nagendra 2007). In this context, our study takes the Dudh Koshi hydropower project as a case study to demonstrate how these existing mechanisms can be strengthened by explicitly integrating forest habitat connectivity into EIA process. We propose a framework that links impact assessment with reforestation measures, ensuring that restoration efforts not only replace lost forest cover but also contribute to maintaining and enhancing connectivity. Specifically, we (i) assessed the extent of forest habitat fragmentation caused by the proposed hydropower project on the Dudh Koshi River in Nepal by modelling structural habitat connectivity before and after construction, and (ii) used the connectivity models to prioritise potential reforestation areas to compensate for connectivity loss and enhance forest habitat connectivity.

## Materials and methods

### Study area

**Fig. 1:**
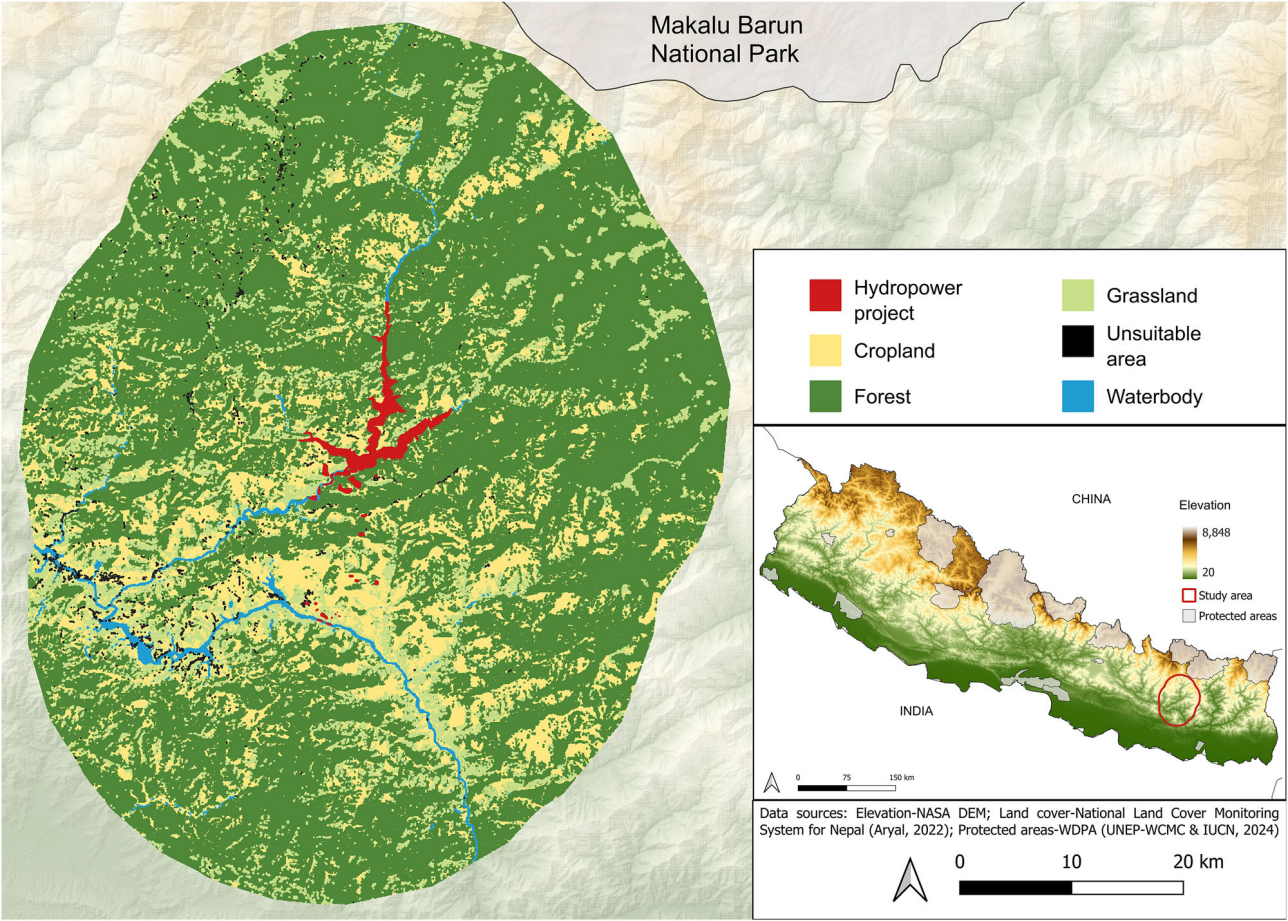
Location of the study area in Nepal and the reclassified land cover used for connectivity modelling. The red polygon indicated the footprint of the proposed hydropower project (NEA 2024)

The framework of this study was applied to a proposed hydropower project located on the Dudh Koshi River in the eastern Nepal, within the Okhaldhunga, Khotang and Solukhumbhu districts (Fig. 1; Dudhkoshi Jalvidyut Company Limited 2024; NEA 2024). The Dudh Koshi River is one of the primary tributaries of the larger Koshi River Basin and is a major tributary of the Sapta Koshi River system. Originating from Imja Peak at an elevation of 6189 m, the Dudh Koshi River is situated within the Himalayan region near Mount Everest. To set an adequate area of analysis for the application of the proposed methodology, a 25 km buffer around the hydropower project footprint was defined, resulting in a total study area of 3792 km^2^ (Fig. 1). This buffer radius was selected considering the median dispersal distance of the common leopard (*Panthera pardus*), a species known to inhabit the area and recognized for its high movement ability. This ensured that study area effectively captured potential impacts also on wide-ranging terrestrial wildlife. The dispersal distance of the common leopard was calculated using the allometric equations of Santini et al. (2013), with home range data from the PanTHERIA database (Jones et al. 2009), a global species-level dataset on mammalian life-history, ecology, and geography, as input. Elevation in the study area ranges between 234 and 3859 m asl, with an average of 1500 m asl, resulting in significant climatic variation from severe cold at higher altitudes to warm, humid conditions at lower elevations. The study area spans three main physiographic zones of Nepal (Heinen and Kattel 1992): the Terai and Inland Terai (below 450 m asl), with most of the area situated in the Middle Hills (between 450 and 3600 m asl) and a small portion extending into the Himalayan zone (above 3600 m asl). The vegetation in these zones is very diverse, ranging from subtropical Sal (*Shorea robusta*) forests with *Dalbergia sissoo* and *Bombax ceiba* in the river plains and lower slopes, to temperate and cold temperate forests with species such as spruce and fir near the tree line, and deciduous species such as oak and rhododendron in the higher elevations (DFRS 2015). Based on a screening on international open database (IUCN 2025), the study area hosts 115 mammals, 654 birds, 66 fish, 67 reptiles, and 27 amphibians. Among these, seven species are classified as Critically Endangered (*Indotestudo elongata*, *Manis pentadactyla*, *Aythya baeri*, *Gyps bengalensis*, *Gyps tenuirostris*, and *Sarcogyps calvus*), and ten are categorized as “Amazing Species” due to their unique ecological or cultural significance (*Tor putitora*, *Elephas maximus*, *Axis porcinus*, *Cuon alpinus*, *Ailurus fulgens*, *Panthera tigris*, *Pardofelis marmorata*) (IUCN 2025).

### Habitat connectivity assessment before and after the construction of the hydropower

#### Land cover maps

In our framework, we assessed the impacts on the structural connectivity of forest habitats, following the methodology outlined by de Sousa Miranda et al. (2021). Land cover map for 2019 was obtained from the National Land Cover Monitoring System for Nepal (Aryal 2022) at a spatial resolution of 30 m, and subsequently resampled using the nearest neighbour technique at a resolution of 60 m due to computational limitations. In addition, to better manage computational complexity, and because some land cover classes were too small, the original classes were combined into five broader categories: Forest, grassland, cropland, water bodies and unsuitable areas (Table 1). This allowed us to focus on the most significant environmental differences. The mixed forest class included the original *forest* and *other wooded land* classes, the water bodies class included *water body* and *riverbed*, and unsuitable areas included land cover classes not suitable for forest habitat connectivity, namely *glaciers*, *snow*, *built-up areas*, *bare soil* and *bare rock* (Table 1). The classes of cropland and grassland remained unchanged from the original ones. Furthermore, given the pronounced altitudinal range of Nepal and our study area, we set an elevation threshold of 3600 m asl, above which areas were classified as unsuitable (i.e., above the tree line; Sharma et al. 2019). This threshold corresponds to the upper tree line and was adopted because our study specifically focused on forest habitat connectivity. After aggregation, the resulting land cover map was used to analyse forest habitat connectivity under current conditions, representing the state of habitat connectivity in the study area before the construction of the hydropower project. After construction, all areas affected by the hydropower project were reclassified in the land cover map as unsuitable areas (Table 1 and Fig. 1); thus, to obtain the land cover used for the after-construction. All spatial analyses were performed in QGIS v.3.28.10 and R v4.3.1 (R Core Team 2023).

**Table 1:** Change in land cover and percentage before and after the construction of the hydropower project

Land cover class	Land cover class reclassified	Before construction		After construction		∆	
		Area (km^2^)	%	Area (km^2^)	%	Area (km^2^)	%
Glacier	Unsuitable areas	11	0.29	34	0.90	+23	+0.61
Snow							
Built-up area							
Bare soil							
Bare rock							
Hydropower project^a^							
Water body	Waterbodies	28	0.74	25	0.66	-3	-0.08
Riverbed							
Cropland	Cropland	1271	33.52	1261	33.25	-10	-0.27
Grassland	Grassland	197	5.19	195	5.14	-2	-0.05
Forest	Forest	2285	60.26	2277	60.05	-8	-0.21
Other wooded land							

Land cover classes from Aryal (2022) were reclassified into broader categories (Land cover class reclassified) and used in this study. ∆ represents the comparison between the before and after construction scenarios

^a^All areas affected by the hydropower project were reclassified in the land cover map as unsuitable areas in the after-construction scenario

#### Connectivity modelling

The final reclassified maps (referred hereafter as before- and after-construction land covers) were used to assess the structural connectivity of forest habitats before- and after-construction. In particular, we used the circuit theory (McRae et al. 2008) to model forest habitat connectivity in our study area using *Circuitscape* package in Julia v.1.8.0 (Hall et al. 2021). *Circuitscape* represents the landscape through which wildlife has to move as a resistance surface, where each element of the landscape has a resistance value: lower resistance values indicate easier movement and higher resistance values indicate obstacles to movement (McRae et al. 2009). Focal nodes represent the sites from which and to which movement flows (i.e. current densities) are to be modelled (McRae et al. 2009). Through the application of circuit theory, areas of higher current density represent greater wildlife movement (Wade et al. 2015). Following de Sousa Miranda et al. (2021), we used the alternative approach to parameterize the resistance surfaces by analysing different resistance profiles representing different landscape permeability of ecologically distinct species. In this way, although simplified, this approach allowed us to assess the structural connectivity of forest habitats and generalize connectivity patterns for multiple species or taxa, rather than focusing on a single species (Fonda et al. 2026). This alternative approach involved creating a set of resistance surfaces where different combinations of resistance values, ranging from 1 (lowest resistance) to 100 (highest resistance), were assigned to each land cover class (de Sousa Miranda et al. 2021). For all resistance surfaces, the forest class was assigned the minimum resistance value of 1, reflecting its optimal habitat suitability for forest wildlife movement. In contrast, unsuitable areas were assigned the maximum resistance value of 100, indicating their highest level of resistance to wildlife movement (Bowman et al. 2020; de Sousa Miranda et al. 2021). Low (10), medium (50) and high (90) resistance values were alternatively assigned to cropland, grassland and waterbodies classes. By considering all possible combinations of these resistance values, a total of 27 resistance surfaces were defined (1 (Forest) × 1 (Unsuitable areas) × 3 (Cropland) × 3 (Grassland) × 3 (Waterbodies) = 27 resistance surfaces) for both the before- and after-construction scenarios. For each of the 27 resistance surface, we ran a model setting (i) the 8-cell neighbourhood rule, and (ii) the pairwise modelling mode (McRae et al. 2013). We randomly located 50 focal nodes in a 16 km buffer surrounding the study area, corresponding to 20% of the maximum length of our study area (Supplementary Materials Figs. S1 and S2; Koen et al. 2014). This approach was used to reduce the effects of artificially high current densities near the focal nodes (Koen et al. 2014). Each model generated a raster of cumulative current density. To obtain a single synthetic map for both the before- and after-construction scenarios, we followed de Sousa Miranda et al. (2021) and summed only the cumulative current density maps that were not highly correlated (i.e., Spearman *ρ* > 0.99).

The change in current density between the two scenarios was quantified for each grid cell as the percentage increase or decrease in current density, with positive values representing an increase in current density and negative values indicating a decrease (Leskova et al. 2022). This was calculated by subtracting the before-construction map from the after-construction map, dividing the resulting change by the before-construction current density, and then multiplying by 100.

A distinctive aspect of this framework is the integration of connectivity loss assessment with a spatial optimisation of reforestation options. While connectivity modelling has been increasingly applied in conservation planning, its explicit use to inform offset design within EIAs remains limited. By combining multi-profile resistance surfaces, consensus current maps and connectivity-based prioritisation, the approach provides an operational tool that can be readily adopted in data-limited contexts, where EIAs often lack quantitative evaluation of terrestrial habitat fragmentation.

### Optimising the selection of reforestation sites

#### Identification of potential areas for reforestation

To identify all areas suitable for reforestation, we compared the most recent land cover data (2019) with the oldest available (2000; Aryal 2022) to detect areas that had experienced deforestation in recent years. Specifically, we first reclassified the 2000 land cover raster using the same classes and pixel resolution as the 2019 land cover (Table 1) and then compared the change per pixel to identify areas that were forested in 2000 but converted to cropland or unsuitable areas by 2019, and areas that remained forested in 2019. Next, based on the results of the after-construction connectivity model, we selected deforested areas with > 20th percentile of current density (de Sousa Miranda et al. 2021).

Finally, we created a hexagonal grid of 10 ha cells (de Sousa Miranda et al. 2021), which reduces edge effects and better aligns grid cells with Euclidean distances (Birch et al. 2007), and overlaid it on the identified forested and deforested areas. Hexagonal cells that overlapped 100% of the forested areas were classified as forested, while those that overlapped at least 10% of the deforested areas were classified as deforested. All adjacent forested hexagonal cells were merged (Tambosi et al. 2014), while deforested cells were no merged.

The procedure above described for the identification of potential areas for reforestation was carried out for the entire study area. After this first assessment, we selected deforested hexagonal cells that overlap, intersect or are within the rivers to obtain a set of potential reforestation areas specifically associated with riparian zones. This targeted analysis was conducted because riparian habitats are among the most significantly impacted by the hydropower project. Enhancing connectivity in these areas is crucial as an offset, given the ecological importance of riparian zones in supporting biodiversity and maintaining habitat continuity along the river corridor. All the analysis to identify potential areas for reforestation were done in QGIS v.3.28.10.

#### Integral index of connectivity

The Integral Index of Connectivity (IIC) is a widely used metric for assessing the structural connectivity of habitat patches, accounting for their spatial configuration, and quantifies the individual contribution of each patch to maintaining or increasing habitat connectivity (Pascual-Hortal and Saura 2006; Saura and Rubio 2010). The IIC is a binary connectivity index where two habitat patches are considered connected if the distance between them is less than or equal to a predefined threshold, otherwise, they are considered disconnected (Saura and Rubio 2010). This index was used to identify optimal sites for reforestation activities aimed at promoting and enhancing habitat connectivity (Pascual-Hortal and Saura 2006). Following de Sousa Miranda et al. (2021), we calculated the IIC for each potential reforestation area, represented as deforested hexagonal cells, to determine their contribution to improving habitat availability and connectivity, if reforested. Using Conefor Sensinode 2.6 (Saura and Torné 2012), we simulated the reforestation of each deforested hexagonal cell, converting them into forested areas (function in Conefor Sensinode 2.6 “node to add”) (Tambosi and Metzger 2015), and considered forested areas as habitat patches, using the current density from the after-construction scenario divided by patch area as the patch attribute. The importance of each deforested cell was quantified using the variation of the IIC (varIIC), a metric that captures the relative chance in connectivity resulting from reforesting a specific cell. Higher varIIC indicated a greater importance on connectivity, helping to prioritise cells for reforestation efforts to achieve maximum benefit. Furthermore, as IIC evaluates connectivity by considering the presence or absence of connections between hexagonal cells based on distance thresholds, we performed the IIC calculations using different distance thresholds (500 m, 1 km and 2 km; within the range of thresholds commonly applied in connectivity studies, e.g. Laita et al. 2011; de Sousa Miranda et al. 2021). These thresholds were selected to simulate species movements with varying dispersal capabilities. After estimating the area of forest loss caused by the project construction (i.e. 8 km^2^), reforestation scenarios were considered by selecting a number of hexagonal cells for each dispersal distance (i.e. 80 hexagonal cells with highest varIIC for each dispersal distance threshold), corresponding to the total estimated area of forest removed.

This analysis was applied to the identified deforested areas throughout the study area to ensure that all potential reforestation sites were assessed for their contribution to improving overall habitat connectivity. Additionally, an alternative approach was also taken, focusing exclusively on riparian deforested areas. These were evaluated using the same method described above, including the calculation of varIIC under different dispersal distances (500 m, 1 km and 2 km). By focusing on riparian zones as a subset of the study area, we were able to assess their specific contribution to improving riparian habitat connectivity and to integrate a targeted restoration strategy to compensate for the impacts of a water-related project such as hydropower.

## Results

### Forest habitat connectivity loss and changes

The land cover of the study area in 2019 is predominantly forested (60%), with forests being more extensive and continuous in the northern region, while in southern they are more fragmented and interspersed with agricultural land. Croplands (33%) and unsuitable areas, including built-up areas, are primarily located around the water bodies (Fig. 1 and Table 1). The proposed hydropower project is expected to occupy an area of 23 km^2^, currently dominated by forests and croplands, with a smaller part covered by grasslands and water body (Table 1). According to the used land cover, a total of 8 km^2^ of the forest class will be lost due to the construction of the hydropower project (Table 1).

**Fig. 2:**
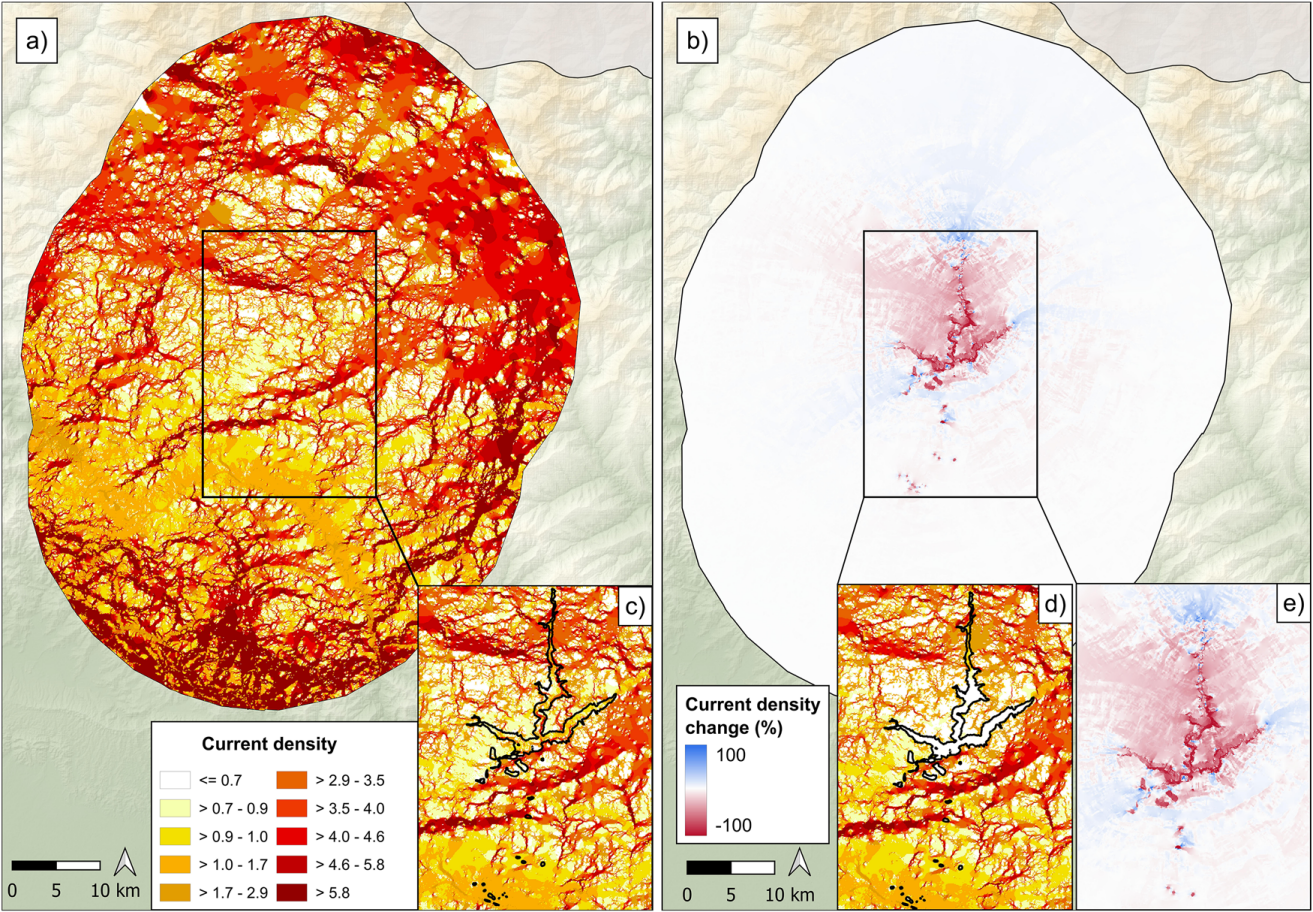
Outputs of connectivity models generated using *Circuitscape* in the before construction scenarios (**a**), and the percentage of current density change between the before and after construction scenarios (**b**); **c** and **d** provide zoomed-in view of the connectivity models within the project area for the before construction and after construction scenarios, respectively. Panel **e** highlights the zoomed-in of the percentage change in current density within and around the project area

As the study area is sparsely populated and predominantly forested, all connectivity models identified numerous corridors, with only minor differences throughout the area (Fig. 2a). In the northern region, the higher in term of mean elevation value, the corridors are wider and exhibit a more dispersed current density due to the presence of contiguous forests. In contrast, the southern region shows a more concentrated current density, resulting in narrower and more defined corridors (Fig. 2a). In the project area, the current density in the before-construction scenario is generally high but is expected to be entirely lost in the after-construction scenario (Fig. 2a, c, d). The construction of the hydropower project will have major impact on habitat connectivity, resulting in the complete loss of corridors within project area and a substantial decrease beyond the project footprint (Fig. 2). Indeed, reduced current densities of around 10% were observed up to 15 km from the project footprint. Conversely, an increase in current density was observed in the northern and southern surrounding areas of the project, reflecting the adjustment of current to find alternative routes around the project (Fig. 2b, e).

### Reforestation priorities

**Fig. 3:**
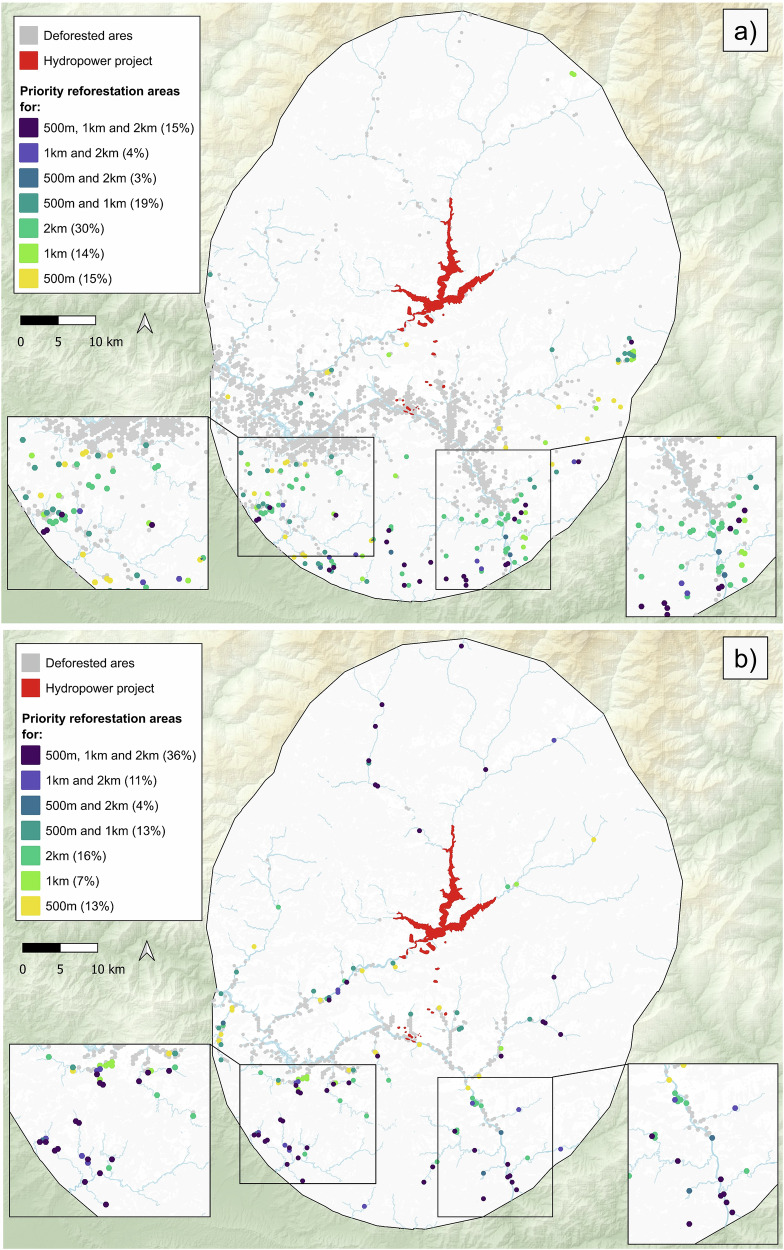
Maps showing the locations of deforested areas identified comparing land cover between 2000 and 2019, along with the priority areas for reforestation activities based on different dispersal distances: 500 m, 1 km and 2 km. **a** Represent the reforestation priorities in the entire study area; **b** represent the priorities identified by the deforested areas along the rivers

Based on the assessment of land cover change between 2000 and 2019, we identified a total of 94.1 km^2^ of deforested areas within the study area (25 km buffer area from the project footprint). Of these, 41.8 km^2^ had a current density above the 20th percentile in the after-construction scenario (i.e., 0.86), representing all potential sites for reforestation activities. These areas were converted into hexagonal cells, resulting in 2827 hexagons throughout the study and 560 hexagons that overlapped, intersected or were within the rivers, i.e. those related to riparian zones. The varIIC ranges for the 80 prioritised hexagons, across the three dispersal distances and for both the entire study area and riparian zone, are presented in Table 2. Overall, priority areas for reforestation across all dispersal distances were predominantly located in the southern part of the study area, where forest fragmentation is more pronounced (Fig. 3). When considering the entire study area, most of the areas were prioritised for only one dispersal distance, with only 15% being prioritised for all dispersal distances (Fig. 3a). Specifically, 30% of the hexagons were prioritised exclusively for the largest dispersal distance (2 km), 14% for the intermediate distance (1 km) and 15% for the smallest dispersal distance (500 m) (Fig. 3a). In contrast, when focusing exclusively on riparian deforested areas, the priority classifications showed greater overlap among dispersal distance thresholds. Specifically, 36% were prioritised for all dispersal distances, while 13% were prioritised only for the largest dispersal distance (2 km), 7% for the intermediate distance (1 km) and 16% for the smallest dispersal distance (500 m) (Fig. 3b).

**Table 2:** Minimum and maximum varIIC values for the three distance thresholds calculated for the 80 prioritized hexagons, i.e. priority reforestation areas, considering both the entire study area and along rivers

Distance thresholds	Entire study area		Along rivers	
	VarIIC min	VarIIC max	VarIIC min	VarIIC max
500 m	242	1737	34	452
1 km	494	4236	49	1774
2 km	4877	126,698	327	17,105

## Discussion

In this study, we proposed a framework for explicitly including forest habitat connectivity into Environmental Impact Assessments (EIAs) to evaluate the impact and guide reforestation efforts as an offset for land-consuming projects. We applied this framework to a real-case hydropower project proposed on the Dudh Koshi River in Nepal, where construction is expected to result in the loss of 23 km^2^ of land, including 8 km^2^ of forest. The framework comprised a first assessment of the change and loss of forest habitat connectivity, comparing before- and after-construction scenarios and, secondly, a spatial prioritisation of reforestation sites to enhance connectivity for species with different movement abilities as an optimised offset. An innovative component of this study lies in linking the quantification of connectivity loss with a structured prioritisation of reforestation offsets. Few EIAs currently incorporate landscape-scale connectivity metrics, and even fewer couple impact assessment with tools capable of identifying where restoration would yield the greatest ecological return. The dual prioritisation approach adopted here, considering both the entire landscape and riparian zones, further enhances the applicability of the framework to hydropower projects, where impacts propagate beyond the reservoir and disproportionately affect riverine habitats. This approach enhances the accuracy of impact assessments and supports the design of targeted compensatory measures, providing a more reliable, quantitative and representative evaluation of the actual impacts while offering a useful way to include connectivity consideration into all stages of EIAs (Bergès et al. 2020).

### Impact on forest habitat connectivity

While hydropower offers low-carbon energy compared to fossil fuels, it poses relevant impacts on loss of forest connectivity, as demonstrated in this study and in previous research (Gibson et al. 2017; Asher and Bhandari 2021). These impacts, along with their spatial extent, should not be underestimated and must be systematically considered into EIAs for hydropower or other renewable energy projects, given their current and future expansion (Bergès et al. 2020; IEA 2024). Renewable energy, including hydro, solar and wind, often require extensive geographical space, which can lead to potential conflicts between energy development and biodiversity conservation goals (e.g., Leskova et al. 2022; Patel et al. 2024).

The application of our framework to the proposed hydropower project enabled us to identify the spatial extent and specific areas most affected by connectivity loss. The construction of the hydropower project will result in the complete loss of forest habitats within the project area, including riparian forests, which have important ecological functions and can serve as corridors for many terrestrial wildlife species (Larsen-Gray and Loehle 2022). Furthermore, due to their specific environmental characteristics and limited distribution across the landscape, these riparian forests are particularly difficult to replace through reforestation efforts (Hughes et al. 2005). Longitudinal (upstream and downstream along the river) and lateral connectivity (movement between riverbanks) of riparian forests are essential for enabling the movement of terrestrial species (Jeong et al. 2018). Particularly in countries with distinct dry and wet seasons, such as Nepal, dry or non-flowing rivers are increasingly recognized as priority ecosystems for the conservation of terrestrial species (Steward et al. 2012; Sánchez-Montoya et al. 2023). Consequently, the loss of these corridors is likely to disrupt both longitudinal and lateral wildlife movements, with relevant ecological implications. Our results further revealed that forest habitat connectivity loss extended well beyond the reservoir area, affecting regions up to 15 km from the project footprint. Similarly, Dorber et al. (2023) demonstrated that hydropower and associated infrastructure impacts on reindeer connectivity extended well beyond the project boundaries. These findings confirm the large-scale impacts of hydropower development on terrestrial habitat and connectivity, highlighting that using only the area occupied by hydropower projects as a metric for estimating terrestrial habitat impacts is overly simplistic and insufficient to capture the broader consequences (Dorber et al. 2023).

### Spatial prioritisation for reforestation efforts

When alternatives within the mitigation hierarchy (IFC 2019) are not feasible, and forest loss is unavoidable, reforestation activities can serve as a critical offset (Cares et al. 2023). Moreover, in many countries, including Nepal, national legislation mandates reforestation following deforestation as a legal requirement (Forest Act, 2019 (2076)). To address the need for reforestation and the impact on forest habitat connectivity, our study applied a method previously used for restoration efforts (e.g., Tambosi et al. 2014; Tambosi and Metzger 2015; de Sousa Miranda et al. 2021) to spatially prioritise the most suitable areas for reforestation to enhance connectivity. Overall, our analysis revealed that most deforested areas are concentrated in the southern part of the study area, particularly along the Sun Koshi River. This pattern indicates relevant human-driven deforestation pressure on riparian habitats over the past 20 years, highlighting an additional stress factor impacting these important ecosystems (Iwata et al. 2003). Our approach to selecting priority areas for reforestation was designed to address two different alternatives: a broad assessment of all deforested areas within the study area, and a targeted assessment of areas considering compensatory reforestation sites close to rivers. Focusing on the entire study area ensures that reforestation efforts improve habitat connectivity on a larger spatial scale, reducing fragmentation and supporting species with different movement abilities across the wider landscape. This comprehensive alternative prioritises deforested areas that could provide the greatest contribution on improving connectivity, regardless of their specific location, and can therefore provide an effective basis for targeted species conservation actions (Li et al. 2017) and broader forest landscape planning (García-Feced et al. 2011). However, this strategy does not specifically target the restoration of riparian forests, which are among the most affected by the proposed hydroelectric project. In contrast, prioritising reforestation efforts along riparian zones allows for the restoration of important habitats and, potentially, for restoring longitudinal and lateral connectivity along waterways. Riparian forests act as natural corridors connecting fragmented habitats, facilitating species dispersal and providing essential ecosystem services (Larsen-Gray and Loehle 2022). However, this targeted approach is limited by the relatively small number of riparian areas available for reforestation, which may constrain its scope.

This dual approach ensures a balanced prioritisation strategy, combining a broad assessment with a more targeted plan for riparian areas. By considering both widespread reforestation opportunities and the specific restoration needs of riparian habitats, the approach aims to optimise the ecological and practical effectiveness of reforestation efforts. The results of the spatial prioritisation for reforestation efforts revealed different patterns and outcomes between the two alternatives. For the entire study area, the prioritised areas were often spatially dispersed, with limited overlap across the different dispersal distances. In contrast, the analysis of the riparian zone showed a more concentrated pattern of priority areas, with greater overlap across dispersal distances, partly due to the smallest number of cells available for reforestation. Importantly, considering multiple spatial scales in such assessments allows for a more comprehensive approach that accounts for species with different movement abilities. Areas identified as priorities across all dispersal distances should be given particular attention, as they have the greatest potential to support connectivity for a wider range of species.

### Limitation of the study and future perspectives

This study achieves the purpose of explicitly integrating forest habitat connectivity into EIAs by quantifying connectivity losses and identifying priorities for reforestation aimed to enhance connectivity. It provides a useful, transferable tool to support impact assessment and offsetting measures in reservoir-based hydropower projects and other land-consuming developments. However, the framework necessarily simplifies the ecological and socio-political complexity of real-world offsetting processes due to its forest connectivity-centred focus.

In terms of methodology, the approach is based on structural rather than functional connectivity. Therefore, it does not take into account species-specific characteristics, habitat requirements or demographic processes (Keeley et al. 2021) and requires validation in the field. While this generalisation increases transferability and reduces data requirements, particularly useful in regions or contexts where data availability is often scarce, it also limits the capacity to fully capture ecological nuances, particularly in areas hosting threatened or specialist species. An interesting development from our study will be the inclusion of species-specific data to inform modelling process and obtain species-specific connectivity assessment (e.g. Leskova et al. 2022). Furthermore, reforestation is not always equivalent to restoring ecosystem functioning, and particularly in riparian habitats, ecological recovery can be slow and uncertain due to altered hydrological regimes and disturbance dynamics (Sweeney et al. 2002; Wohl et al. 2015).

The social and economic dimensions of reforestation are equally important and strongly influence the feasibility of offsetting. In Nepal, the involvement of community forest institutions provides an alternative governance model that has proven effective in combining forest restoration with local livelihoods (Nagendra 2007). However, prioritisation outcomes based purely on connectivity may not always align with local needs or land-use priorities. Thus, stakeholder engagement is crucial to ensuring the feasibility and long-term sustainability of restoration (BBOP 2012). Without such engagement, even ecologically optimal restoration plans may be resisted or fail to be implemented due to competing land uses, limited technical capacity or political constraints.

These considerations emphasise that the use of connectivity as the main criterion for reforestation prioritization should be viewed as one component within a broader offsetting strategy. Our results show that connectivity-based assessments can add transparency and scientific rigor to EIAs, complementing the mitigation hierarchy by improving the design of restoration and offsets. However, effective biodiversity offsetting ultimately requires integrating ecological models with participatory decision-making, socio-economic feasibility analyses, and long-term monitoring to evaluate outcomes (Bull et al. 2013). The proposed framework provides a replicable and cost-effective tool for quantifying forest habitat connectivity loss and informing reforestation priorities, thereby advancing the integration of connectivity into EIA practice. At the same time, its application requires complementary studies, field validation and wildlife monitoring of focal species to enhance robustness and long-term effectiveness. For example, Sales Rosa et al. (2022) demonstrated that combining connectivity modelling with systematic wildlife monitoring can substantially improve the design and evaluation of biodiversity offset strategies. Similar approaches could enhance the applicability of our framework, particularly in context where threatened species or habitats are of concern. However, in areas with threatened or protected species or sensitive habitats, reforestation activities alone may not be sufficient to offset the impacts of habitat degradation and connectivity loss. In such cases, intervention should be limited to exceptional circumstances, and projects must prioritise avoidance and minimization of impacts before exploring offset options. Overall, a precautionary and context-specific approach is needed to ensure that ecological integrity is not compromised by energy development, while balancing the urgent need for clean energy with biodiversity conservation.

## Supplementary information

Supplementary materials

## Data Availability

No datasets were generated or analysed during the current study.
