# Supplementary material for: A framework for assessing forest habitat connectivity loss and optimising reforestation efforts on the example of a hydropower project
Source: Environ Manage. 2025 Dec 27;76(2):52. doi: 10.1007/s00267-025-02351-7 (PMC12743694; doi:10.1007/s00267-025-02351-7)
Supplement: Supplementary file 1 — Supplementary materials [file 267_2025_2351_MOESM1_ESM.docx]

**Supplementary materials**

**A framework for assessing forest habitat connectivity loss and optimising reforestation efforts on the example of a hydropower project**

Fonda Federica^1,*^, Petrillo Maria^2^, Bacaro Giovanni^1^

^1^ Department of Life Science, University of Trieste, via L. Giorgieri 10, 34127 Trieste, Italy

^2^ Department of Agricultural, Food and Forest Sciences (SAAF), University of Palermo, Viale delle Scienze, 90128 Palermo, Italy

* Corresponding author: Federica Fonda, [federica.fonda@gmail.com](mailto:federica.fonda@gmail.com)

**Figure S1 -** Output of connectivity model generated with *Circuitscape* for the before construction scenario. Black dots represent the 50 focal points randomly distributed within the buffer surrounding the study area.

**
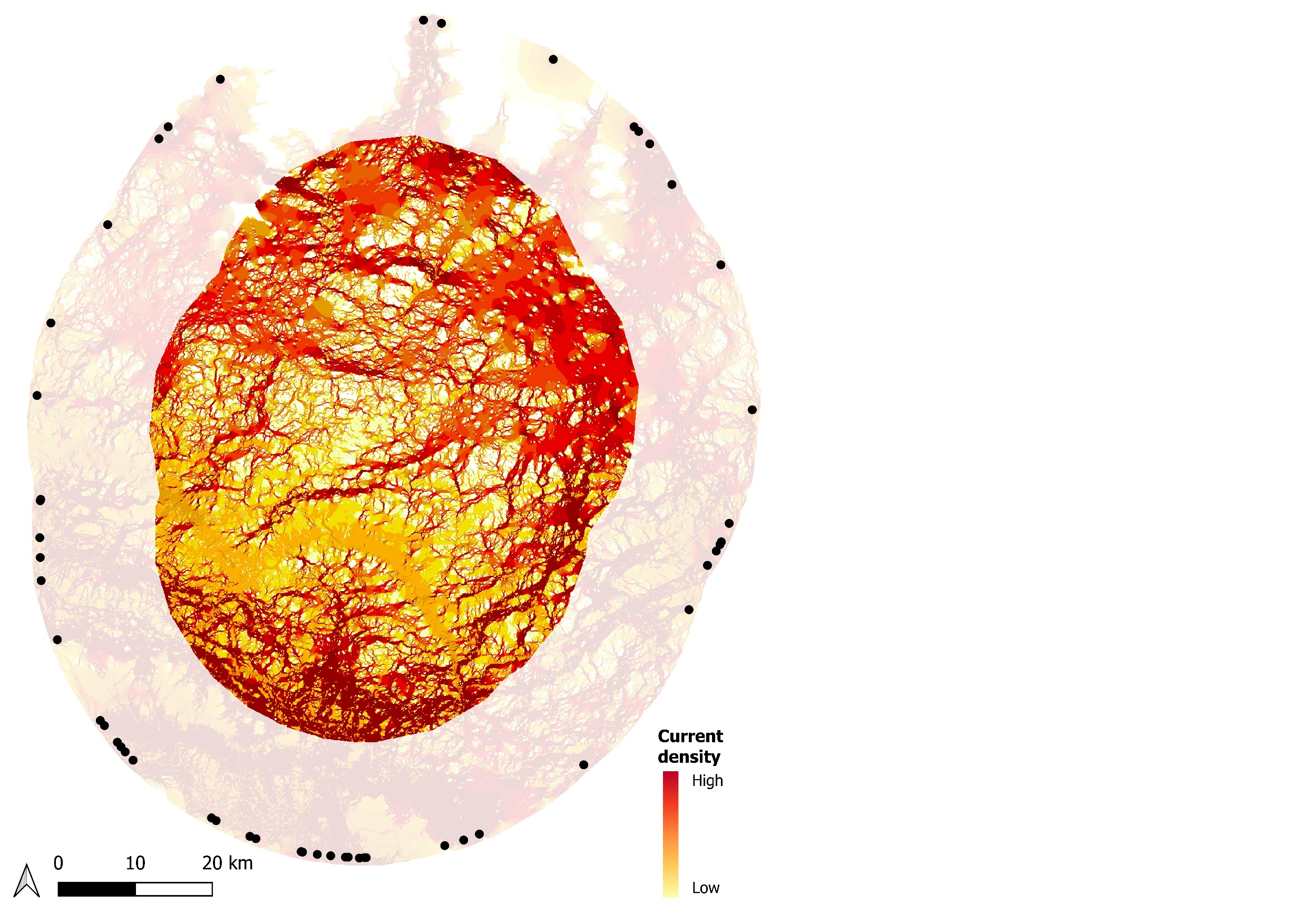
**

**Figure S2 -** Output of connectivity model generated with *Circuitscape* for the after construction scenario. Black dots represent the 50 focal points randomly distributed within the buffer surrounding the study area.

**
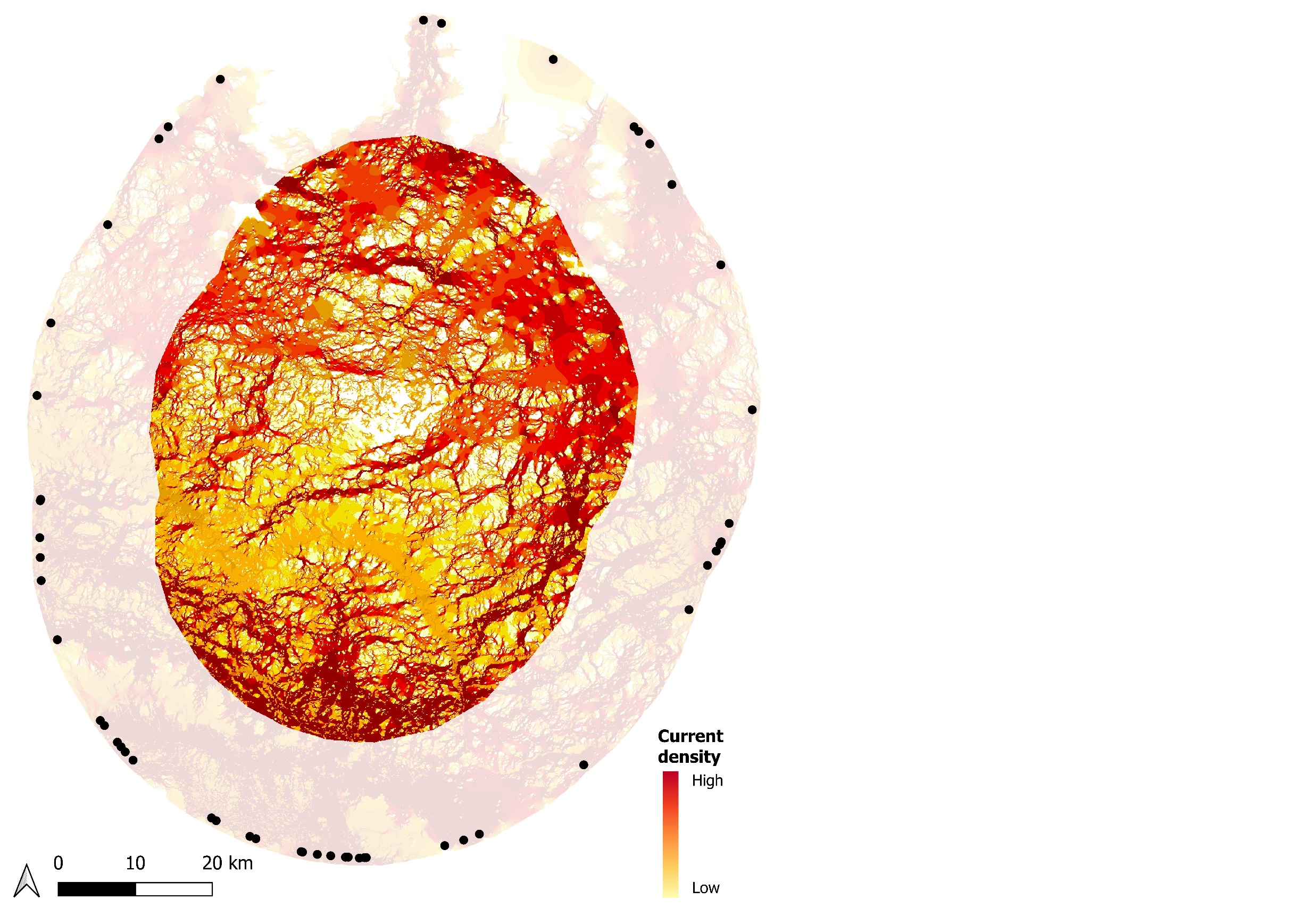
**
